# Evaluation of High-Pressure Processing in Inactivation of the Hepatitis E Virus

**DOI:** 10.3389/fmicb.2020.00461

**Published:** 2020-03-24

**Authors:** Neda Nasheri, Tanushka Doctor, Angela Chen, Jennifer Harlow, Alexander Gill

**Affiliations:** National Food Virology Reference Centre, Bureau of Microbial Hazards, Food Directorate, Health Canada, Ottawa, ON, Canada

**Keywords:** Hepatitis E virus, high-pressure processing, infectivity assay, droplet-digital RT-PCR, pork product

## Abstract

Hepatitis E virus (HEV) causes acute hepatitis with approximately 20 million cases per year globally. Based on genetic diversity, HEV is classified into different genotypes, with genotype 3 (HEV-3) being most prevalent in Europe and North America. The transmission of HEV-3 has been shown to be zoonotic and mainly associated with the consumption of raw or undercooked pork products. Herein, we investigated the efficacy of high-pressure processing (HPP) in inactivation of HEV-3 using a cell culture system. HPP has been indicated as a promising non-thermal pathogen inactivation strategy for treatment of certain high-risk food commodities, without any noticeable changes in their nature. For this purpose, we treated HEV-3 in media with different conditions of HPP: 400 MPa for 1 and 5 min, as well as 600 MPa for 1 and 5 min, at ambient temperature. All four HPP treatments of HEV in media were observed to result in a 2-log reduction in HEV load, as determined by the amounts of extracellular HEV RNA produced at 14-day post-infection, using the A549/D3 cell culture system. However, application of the same treatments to artificially contaminated pork pâté resulted in 0.5 log reduction in viral load. These results indicate that the efficacy of HPP treatment in the inactivation of HEV-3 is matrix-dependent, and independent of maximum pressure between 400 and 600 MPa and hold time between 1 and 5 min. Based on the obtained results, although the HPP treatment of pork pâté reduces the HEV-3 load, it might not be sufficient to fully mitigate the risk.

## Introduction

Hepatitis E virus is a single-stranded RNA virus with positive polarity belonging to the *Hepeviridae* family (Smith et al., 2014). The HEV genome has three open reading frames (ORFs): ORF1 encodes a long non-structural polyprotein with multiple functions; ORF2 encodes the viral capsid protein; and ORF3 encodes a small phosphoprotein with structural and non-structural functions (Panda and Varma, 2013). The *Hepeviridae* contain two genera *Orthohepevirus* and *Piscihepevirus*, which infect a wide range of vertebrate hosts (Primadharsini et al., 2019). Four genotypes (HEV-1, HEV-2, HEV-3, and HEV-4) of the species *Orthohepevirus* A are associated with human illness. HEV−1 and HEV−2 are restricted to humans and are prevalent in regions with poor water sanitation, such as the developing countries of Asia, Africa, South and Central America (Hartl et al., 2016; Pisano et al., 2018). On the other hand, HEV-3 and HEV-4 are considered to be zoonotic pathogens as they have a much wider range of mammalian hosts including, among others, domestic and wild swine and ruminants (Park et al., 2016; Sooryanarain and Meng, 2019). Hepatocytes have been identified as the primary sites of HEV replication, but the virus has been detected in other tissues such as epithelial cells of the small intestine, placenta, and muscle (Williams et al., 2001; Bose et al., 2014; Salines et al., 2019).

Clinical manifestation of HEV can vary depending on virus genotype and the host. It is generally believed that the majority of HEV infections are subclinical (Murrison and Sherman, 2017). In symptomatic cases, HEV most commonly presents as a self-limiting, acute infection (Hartl et al., 2016; Chauhan et al., 2019). However, chronic HEV infection can occur after infection with HEV-3, and possibly HEV-4, specifically in immunosuppressed patients, such as human immunodeficiency virus patients or those receiving immunosuppressing treatment (Fujiwara et al., 2014; Kamar et al., 2015). In recent years, the incidence rate of HEV-3 infection has increased in industrialized countries, likely through zoonotic exposure (Van der Poel et al., 2018). Due to the lack of surveillance data, the actual HEV incidences and fatalities per country are often unknown, and therefore the true burden of HEV disease remains unclear (King et al., 2018; Van der Poel et al., 2018).

Multiple lines of evidence indicate that infection with HEV-3 is common among domestic swine in developed countries (reviewed in Salines et al., 2017); however, HEV-3 viremia in swine does not cause any noticeable clinical symptoms (Krog et al., 2019; Motoya et al., 2019). HEV-3 infection of domestic swine can potentially result in contamination of pork products. The reported prevalence of contaminated pork products varies from <1% to >50%, depending on the region and the tested commodity (reviewed in Salines et al., 2017). In a previous study conducted by our laboratory it was observed that 10.5% of sampled raw pork livers, and 47% of the sampled commercial pâté, marketed in Canada, were positive for HEV RNA (Mykytczuk et al., 2017). Because of this high prevalence, efficient strategies to inactivate HEV in ready-to-eat pork products should be considered in order to prevent foodborne HEV infection.

High pressure processing (HPP) is a “non-thermal pasteurization” technique, which can inactivate foodborne pathogens within certain commodities such as ready-to-eat meats and fruit juices to increase their shelf life or improve safety (Kingsley, 2013). It is generally believed that high-pressure treatment denatures the viral capsid proteins and therefore incapacitates the infection virions from attachment and penetration to the host cells (Kingsley, 2013; Emmoth et al., 2017). However, due to the lack of reliable infectivity assays, most HEV inactivation studies to date have been limited to using surrogate viruses (Cook et al., 2017; Emmoth et al., 2017). Recently, successful replication of HEV-3c strain 47832c (GenBank accession No. KC618403), in A549/D3 cells was demonstrated by Johne et al. (2016; Schemmerer et al., 2016). This system has been employed to study the temperature sensitivity of HEV (Schemmerer et al., 2016), and inactivation by silvestrol (Glitscher et al., 2018), demonstrating a potential for this system to be used in other HEV inactivation studies. Herein, we describe the employment of this HEV infectivity assay to examine the effect of HPP treatment on HEV infectivity in both cell culture media and ready-to-eat pork pâté.

## Materials and Methods

### Cells and Viruses

A549/D3 human lung carcinoma cells were kindly provided by Dr. R. Johne (German Federal Institute for Risk Assessment, Berlin), as two cell lines, with and without persistent infection with HEV genotype 3C strain 47832c. Both A549/D3 cell lines were cultured in growth medium composed of Minimum Essential Media (MEM) (Gibco, MA, United States), supplemented with 1% non-essential amino acids, 1% glutamine, 0.5% gentamicin, and 10% fetal bovine serum (FBS) (Gibco, MA, United States).

The optimal cell density of A549/D3 cells per well was determined to be 4 × 10^4^ cells per well of a 96-well plate, with 100 μl/well of growth medium. The plate was then incubated for 2 days at 37°C and 5% CO_2_. Growth medium was replaced with 100 μl of fresh media and the cells were incubated under the same conditions for another 3 days, until the infectivity assay was performed.

Virus particles of HEV-3 47832c were prepared for experiments by removing supernatant from a culture of the persistently infected A549/D3 cell line. The concentration of viral genomes in the supernatant was determined by digital droplet RT-PCR (ddRT-PCR) as described below, and the concentration of the supernatant adjusted by dilution in fresh growth medium as required.

### HEV Infectivity Assay

The HEV infectivity assay using the A549/D3 cell line, developed by Johne et al. (2014) (Edgar, 2004), was used to enumerate the infectious particles of the HEV-3 virus in samples. After 5 days of incubation of A549/D3 cells at 37°C and 5% CO_2_, the growth medium was removed and each well was washed twice with 200 μl of PBS. Cells were infected using 100 μl of the virus suspension. After incubation for 1 h at room temperature, the virus was removed and cells were washed twice with 200 μl of PBS. Growth medium with 5% FBS was added to each well in 200 μl amounts and cells were incubated for 7 days at 34.5°C and 5% CO_2_. The growth medium was replaced with 200 μl fresh growth medium with 5% FBS and incubated for another 7 days in the same conditions, for a total of 14 days. Growth medium was collected after 14 days and was frozen at −80°C until processed for RNA extraction.

### Sample Preparation for HPP Treatment

Sterile polyethylene (PE) tubes (Tygon^®^) 1.5 cm in length were filled with 200 μl of cell growth medium containing 2 × 10^6^ RNA copies of HEV-3 strain 47832c and heat-sealed. Triplicate tubes were prepared in sets for each treatment duration (untreated control, 1, or 5 min), for a total of nine tubes. The tubes for each treatment then placed in PE bags containing 10% bleach, to inactivate viral particles in the event of leak or rupture from the primary container. The sample bags were then heat-sealed, while minimizing air bubbles in the bleach solution. Prepared sample bags were stored on ice until the HPP treatment.

Pork pâté samples were prepared from commercial product obtained from a local grocery store, in Ottawa, ON, Canada (containing 8% sodium, 27% fat, and 10% protein). Individual samples of 2 g were weighed out to prepare triplicate samples. An uninoculated pâté sample was retained as a negative control. Samples were inoculated with 250 μl of cell culture medium containing approximately 1 × 10^7^ RNA copies, which was spread over the entire surface area of the sample. Inoculated samples were dried for 10 min in a biosafety cabinet at ambient temperature (i.e., 22°C), to ensure the virus suspension is absorbed by the matrix prior to being placed in individual PE bags, which were heat-sealed with minimal air space. Triplicate samples for each treatment duration (1 and 5 min) were then placed in a second PE bag containing 10% bleach, and stored on ice prior to HPP treatment. Untreated positive controls were inoculated as described before in triplicate, but left at room temperature for the duration of the treatment.

### HPP Treatment

High-pressure processing was performed using a high-pressure pilot unit manufactured by Dustec Hochdrucktechnik GmbH (Wismar, Germany), with a 1-L pressure vessel and water as the pressure medium. The rate of pressurization was 10 MPa/s and the rate of depressurization was −20 MPa/s. Sample packages were pressurized to 400 or 600 MPa with a hold time at maximum pressure of 1 or 5 min. As determined by three thermocouples inside the pressure vessel, the temperature of the pressure medium was initially 24.0°C [standard deviation (SD) 0.3°C, *n* = 4]. Adiabatic heating during pressurization resulted in an average temperate increase of 8.2°C (SD 0.1°C, *n* = 2) when pressurized to 400 MPa and 12.9°C (SD 0.1°C, *n* = 2) when pressurized to 600 MPa.

### Virus Extraction

The procedure used to extract HEV from pork pâté samples post-HPP treatment was adapted from the ISO 15216-1:2017 “soft fruits and salad vegetables” method (Anonymous, 2017). This method allowed for precipitation of intact viral particles, which could then be used for infectivity assays. Briefly, the pâté samples were transferred to stomacher bags with a filter compartment and 16 mL of Tris Glycine Beef Extract (TGBE) was added, respectively. No pectinase treatment was performed. The stomacher bags were then incubated on a rocking plate at room temperature for 20 min. The resulting suspension was centrifuged at 10,000 × *g* for 30 min at 4°C. The supernatant pH was balanced using approximately 110 μl of 12 N HCl. 5× PE glycol 6000 (PEG)/NaCl of 1/4 volumes of the weight of each sample was added to each tube and the samples were incubated on ice on a rocking plate for 1 h. Post incubation, samples were centrifuged at 10,000 × *g* for 30 min at 4°C and the supernatant was discarded. The pellets containing the virus particles were suspended in 500 μl PBS and stored at −80°C until required for RNA extraction or the infectivity assay.

The viral extraction efficiency was calculated by comparing the number of viral RNA copies recovered from the untreated inoculated pate with the number of RNA copies used to inoculate the pâté (Mykytczuk et al., 2017).

### Determining the Limit of Quantification

In order to determine the limit of quantification of the infectivity assay, cell culture-adapted HEV-3 strain 47832c at 10 concentrations from 5 × 10^2^ to 1 × 10^6^ RNA copies per well (in 100 μl media) were used to infect A549/D3 cells in triplicate experiments (Johne et al., 2016; Schemmerer et al., 2016). The media was replaced at 1 h, and 7 days post-infection. The infected cells and negative control cells, which were not exposed to HEV-3 strain 47832c, were cultured for 14 days. The media supernatant was then collected and the extracellular HEV RNA levels were analyzed by droplet-digital RT-PCR (ddRT-PCR).

### RNA Isolation and Quantification

The Viral RNA Mini Kit (Qiagen, Mississauga, ON, Canada) was used to extract RNA from the collected infectivity assay growth medium. Quantification of recovered RNA was conducted as previously described using Bio-Rad droplet digital PCR (ddRT-PCR) technology (Mykytczuk et al., 2017; Nasheri et al., 2017).

### DNA Sequencing

Immediately after HPP treatments, the viral solutions were subjected to RNA extraction and Sanger sequencing as follows. Conventional RT-PCR was carried out using the HEV-11 primers (Johne et al., 2014), which amplifies the region between the positions 5468-6018 of the HEV-3 strain 47832c (550 bp) and the Qiagen one-step RT-PCR kit according to the manufacturer’s instructions, followed by gel-extraction of the product of expected size using QIAquick gel-extraction kit (Qiagen, Mississauga, ON, Canada) according to manufacturer’s instructions. Gel-purified RT-PCR products were sequenced directly using the BigDye^®^ terminator v 3.1 DNA sequencing kit (Applied Biosystems, Foster City, CA, United States) according to manufacturer’s instructions (18 μl BDT reaction and 2 μl of DNA template). Fluorophore-labeled reactions were purified using the Wizard^®^ MagneSil^®^ Sequencing Reaction Clean-up System (Promega, Madison, WI, United States). Samples were sequenced in both directions using the HEV11F 5′-CGGCAGTGGTTTCTGGRGTGAC-3′, HEV11R 5′-GTAATAGAGTTCATRTCAACAGA-3′ primers, and a 3130xl Genetic Analyzer (ThermoFisher Scientific). HEV-positive sequences were determined by querying NCBI BLAST and edited using BioEdit (Ibis Biosciences, Carlsbad, CA, United States).

Multiple sequence alignments were performed using both the Multiple Sequence Comparison by Log-Expectation (MUSCLE) (Edgar, 2004) and Clustal W (Larkin et al., 2007) included in the MEGA6 software (Tamura et al., 2013). The sequences obtained in this study have been deposited in GenBank under Accession Numbers MN535994–MN535997. The genetic codes were translated into protein sequences using the feature embedded in the MEGA6 software.

### Data Analysis

All experiments were performed in triplicate. Statistical analysis was performed by Microsoft Excel 2016. Paired Student’s *t*-test was conducted to obtain *P*-values.

## Results

### Limit of Quantification

**FIGURE 1:**
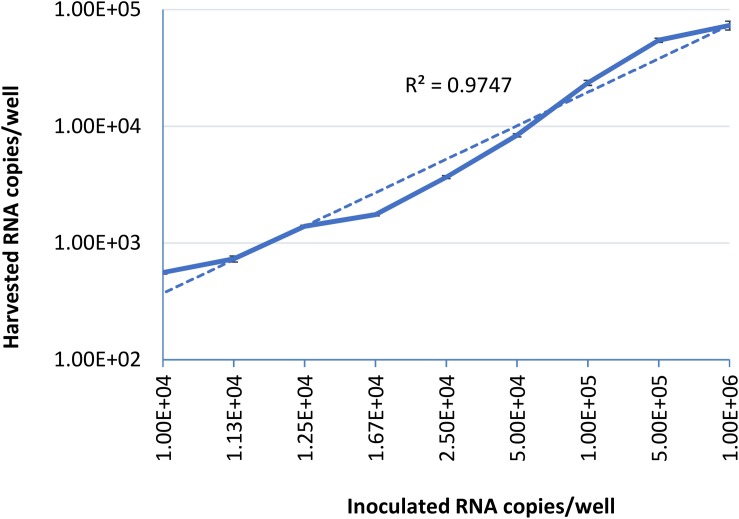
Serial dilutions of HEV-3 strain 47832c were inoculated in triplicates onto A549/D3 cells. The HEV genome copy numbers in the supernatant was quantified at 14 d.p.i. using ddRT-PCR, and the mean copy number and the standard deviation (error bars) of three replicates each are shown. Correlation coefficient (*R*^2^) between the inoculated HEV-3c strain 47832c (genome copy number) and the harvested extracellular HEV (genome copy number) 14 d.p.i. in A549/D3 cells is demonstrated.

To examine the limit of quantification for this infectivity assay, serial dilutions of HEV-3 strain 47832c were inoculated in triplicate onto A549 cells as described above. The HEV genome present in the supernatants of the cell cultures was quantified by ddRT-PCR at 14 days post infection (d.p.i), because it has been demonstrated that the HEV genome copy numbers in the supernatant reach to a plateau by day 14 after the inoculation (Johne et al., 2014). The correlation between the inoculated HEV genome copy number and the harvested genome copy number at 14 d.p.i is shown in Figure 1. The relationship between the two is linear over the range studied with a *r*^2^ value of 0.9823, this demonstrates that the amount of harvested HEV RNA is directly correlated to the amount of HEV inoculum. The limit of quantification by this method was determined to be 1 × 10^4^ RNA copies per well (100 gc/μl) of the inoculated virus, and inoculation with titers below this amount did not reliably and reproducibly yield quantifiable progeny virus at 14 d.p.i. These data also suggest that the ratio between the inoculated genomes and harvested extracellular genomes at 14 d.p.i. is 10.2 ± 4.8 to 1 (Supplementary Table 1).

### HEV Inactivation in Cell Culture Media

**FIGURE 2:**
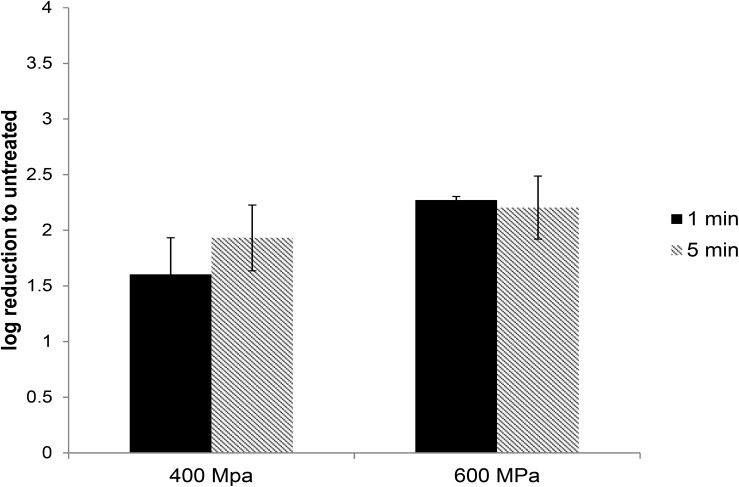
The effect of HPP treatment on HEV-3c strain 47832c in cell culture media. The samples containing 2 × 10^6^ RNA copies were treated at 400 and 600 MPa for 1 and 5 min at ambient temperature in triplicates and were inoculated onto A549/D3 cells. The HEV genome copy numbers in the supernatant were quantified at 14 d.p.i. using ddRT-PCR. The effect is shown in comparison to the untreated viral stock. The standard deviation (error bars) of three replicates is shown.

In commercial food processing, HPP is applied to meat products with pressures typically ranging between 400 and 600 MPa for 1–10 min (Hugas et al., 2002). To determine the role of pressure and hold time on the inactivation of HEV by HPP, HEV-3 strain 47832c, in cell culture media, was treated at pressure levels of 400 and 600 MPa for 1 and 5 min starting at 24°C. The undiluted and 1:10 diluted HPP-treated viral solutions, along with untreated controls, were used to infect A549/D3 cells in duplicate as described above. The decrease in infectious HEV particles was determined by comparing the reduction in HEV RNA at 14 d.p.i. in HPP-treated samples with the untreated controls. As shown in Figure 2, reductions of 1.6 ± 0.33 and 1.93 ± 0.29 log in infectious viral RNA genomes were observed for the samples that were treated at 400 MPa for 1 and 5 min, respectively. Increasing the pressure to 600 MPa resulted in a slight but not statistically significant increase in viral inactivation; 2.27 ± 0.03 and 2.2 ± 0.28 log reduction for 1 and 5 min treatments, respectively (Figure 2). Neither varying the treatment pressure (400 or 600 MPa) nor the hold time at maximum pressure (1 or 5 min) resulted in statistically significant reductions in the viral inactivation (*P* > 0.1) (Supplementary Table 2).

**FIGURE 3:**
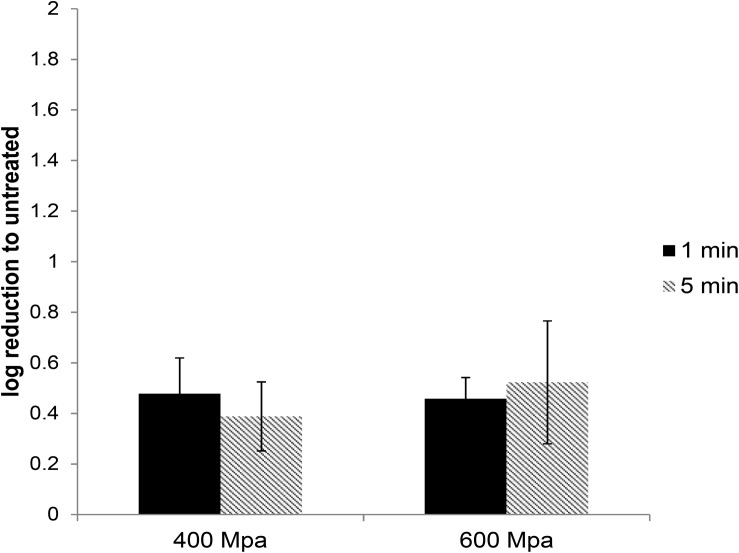
The effect of HPP treatment on HEV-3c strain 47832c in ready-to-eat pork pâté. The samples containing 4 × 10^7^ RNA copies of HEV were treated at 400 and 600 MPa for 1 and 5 min at ambient temperature in triplicates and were inoculated onto A549/D3 cells. The HEV genome copy numbers in the supernatant were quantified at 14 d.p.i. using ddRT-PCR. The effect is shown in comparison to the untreated but inoculated samples. Error bars represent standard deviation.

### Examining Amino Acid Variation in the Capsid Protein

**FIGURE 4:**
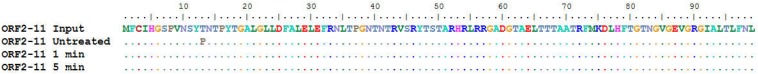
Amino acid sequence alignment of the N domain of the capsid protein (ORF2). The sequences for input, the untreated, treatment at 600 MPa for 1 min hold time and 600 MPa for 5 min hold time were aligned using Mega6 software and translated into amino acid sequences using BioEdit software.

The HEV capsid protein consists of 660 residues and 4 main structural and functional domains; N, S, M, and P (Guu et al., 2009). Potentially, viral particles which retain infectivity following HPP treatment may constitute a subpopulation with a mutation which stabilizes the structure of the capsid protein, which are essential for interaction with host cell receptors. It would be expected that if such mutation existed it would predominate in the viral particles which retain infectivity after HPP treatment. To examine whether the capsid protein of the viruses that survived the HPP treatment is different from that of the input or the untreated viruses, we compared the amino acid sequence of the partial capsid protein encompassing the N and S domains of the viruses treated with 600 MPa for 1 min hold time and 600 MPa for 5 min hold time with the input and untreated viruses. As shown in Figure 4, no synonymous change was observed between the treated and untreated viruses within the sequenced range.

### HEV Inactivation in Ready-to-Eat Pork Pâté

In order to investigate whether food matrices can protect from or potentiate the inactivation of HEV by HPP, experiments were conducted with a high-risk ready-to-eat pork product, artificially inoculated pork pâté. HEV was extracted using the modified ISO15216-1 method and the HEV recovery rate from the pâté samples was determined by the ratio between the extracted viral genomes from the untreated positive controls to the inoculated HEV RNA copies, and it ranged from 3 to 4.6% with an average of 3.7 ± 0.6%.

High-pressure processing treatment at 400 or 600 MPa for up to 5 min did not cause any noticeable change in the appearance of pâté samples (Supplementary Figure 1). The extracted HEV was used to infect A549/D3 cells. At 14 d.p.i. the media was harvested and examined for the presence of viral RNA using ddRT-PCR. The effect of the HPP treatment was determined by comparing the viral load in treated samples against the untreated samples. HPP treatment of pork pâté at 400 and 600 MPa for 1 and 5 min, resulted in significantly lower reductions in infectious HEV than observed in cell culture media. At 400 MPa the reduction in infectious HEV was only 0.48 ± 0.14 and 0.46 ± 0.13 log, and at 600 MPa 0.39 ± 0.08 and 0.52 ± 0.24 log, respectively, for 1 and 5 min treatments (Figure 3). As observed in culture media, no significant difference in virus inactivation was observed between 1 and 5 min treatment at the same pressure (*P* > 0.1), also increasing pressure to 600 from 400 MPa did not result in increased HEV inactivation in pork pâté (*P* > 0.1) (Supplementary Table 3).

## Discussion

Development of a cell culture for HEV, based on HEV strain 47832c replication in A549/D3 cells allowed for investigation of HEV replication and inactivation (Johne et al., 2016; Schemmerer et al., 2016). Herein, we employed this system to examine the effect of HPP in inactivation of HEV. For this purpose, we first determined the limit of quantification for this system for production of extracellular HEV RNA at 14 d.p.i. and observed that inoculation with less than 4 log of HEV strain 47832c RNA does not reproducibly lead to productive HEV infection. This finding is consistent with Schemmerer et al. (2016) observations that inoculation of A549/D3 with titers less than 5.6 × 10^3^ RNA copies does not lead to productive HEV infection examined by focus forming unit assays.

In order to investigate HEV inactivation by HPP treatment, the untreated and treated virus stocks were used to infect the A549 cells and the infected cells were examined for the production of progeny virus in the culture supernatant. Using this system, we demonstrated that an approximately 2-log reduction in viral load can be accomplished by treatment of HEV in media at 400 MPa with a 1 min hold time. Increasing the pressure to 600 MPa or the hold time to 5 min did not have any significant effect on the reduction of viral load. However, HEV in artificially contaminated pâté was protected from HPP treatment, as the reduction in infectious HEV RNA genomes was <0.5 log.

This study quantifies the inactivation of HEV following HPP treatment. HEV response to 500 MPa for 15 min has been previously investigated with RT-qPCR viability markers (PMAxx and platinum chloride, PtCl4-RT-qPCR), but was only able to report the presence of intact viral particles post treatment (Randazzo et al., 2018). The inactivation by HPP of other foodborne viruses, including norovirus, hepatitis A virus (HAV), and surrogates for foodborne viruses has been investigated (Kingsley et al., 2002; Kingsley, 2013; Lou et al., 2015; Lou et al., 2016). The sensitivity of specific viruses to high-pressure treatment can vary significantly. Kingsley et al. (2002) reported that HAV in cell culture media under a 5 min hold period was stable at pressures up to 300 MPa, but inactivation increased with increasing pressure between 300 and 450 MPa, to a maximum of 6 log reduction. In contrast, for feline calicivirus (FCV) (a surrogate for norovirus) 3 log reductions were observed at 200 MPa and no infectious particles were recovered following treatment at 275 MPa (Kingsley et al., 2002). Inactivation of norovirus suspended in buffer has been reported at pressures in excess of 200 MPa (5 min hold, 4°C), but the sensitivity to pressure was variable between the four strains studied, with the most sensitive strain reduced by 4 log at 600 MPa and the least sensitive by only 1 log under the same conditions (Lou et al., 2016). Therefore, it can be concluded that the inactivation efficiency of HPP depends on the studied virus and its surrounding matrix.

Hepatitis E virus is a quasi-enveloped virus (Qi et al., 2015), and the presence of a lipid envelope may have a protective role. If so, the impact of HPP on HEV-3 in media could potentially be enhanced in the presence of membrane disrupting molecules, such as bile acid salts.

The sequence analysis of partial capsid protein revealed that there is no amino acid change between the treated and untreated viruses within the N and S domain. Nevertheless, to determine whether the capsid of the surviving viruses is different from the untreated viruses, full capsid sequence analysis is required. In this study, our attempts to retrieve the full capsid sequence from the treated samples were not successful. Although we cannot rule out the possibility of reversion of mutations during the culture period (14 days).

In this study, we observed that HEV in pork pâté was protected from HPP treatment, compared to HEV in cell culture media. The protective effect of the food matrix against viral inactivation by HPP has also been reported by Kniel and coworkers, as they observed that inactivation of murine norovirus, FCV, and HAV by HPP was reduced in tested food matrices compared to virus in cell culture media under the same pressure parameters (Hirneisen et al., 2012). The dependency on the surrounding matrix of the response of bacterial cells to HPP treatment is a well-established phenomenon, with salt concentration, pH, fat content, and the presence of specific molecules reported to affect cell survival (Balamurugan et al., 2018). Similar observations have been made for viruses, with pH, temperature, and solute concentration reported as variables in the response of norovirus to HPP (Lou et al., 2016). The presence of food components has been demonstrated to protect viral capsids from HPP denaturation (Komora et al., 2018). A further demonstration of the challenge in extrapolating studies in model systems to compel foods, a human volunteer study with oysters inoculated with 4 log PFU of norovirus (GI.1. Norwalk) found that a treatment of 400 MPa (5 min hold, 6°C) was insufficient to protect volunteers, though 600 MPa was protective for all volunteers (Leon et al., 2011). The protective effect of food against different inactivation strategies has been studied for various foodborne viruses and their surrogates (reviewed in Cook and van der Poel, 2015; Cook et al., 2016). For example, it has been reported that fat increases the heat-stability of HEV in pork products (Barnaud et al., 2012) as well as hepatitis A virus in skim milk and cream (Bidawid et al., 2000). It has been proposed that food components such as fat, protein, and salt can interact with the viral capsid under pressure, causing protective effects from inactivation by pressure (Hirneisen et al., 2012). However, whether the fat and salt content of the treated matrix affects the structural integrity of HEV and its sensitivity toward pressure needs to be further investigated.

Our data demonstrated that the viral titer post-HPP treatment of pâté under 600 MPa for 5 min reaches to 1.5 × 10^3^ RNA copies in cell culture supernatant. This indicates that replication of virus occurred, and therefore the elimination of HEV infectivity was not complete. In another study, it was shown that the treatment of HEV solution at 500 MPa for 15 min did not result in complete inactivation assayed by using viability markers (PMAxx and platinum chloride, PtCl4-RT-qPCR) (Randazzo et al., 2018). Knowing that the virus is capable of replication (and therefore has the potential to cause illness) is important for interpretation of surveillance and inactivation studies on HEV to inform risk assessment and mitigation (Cook et al., 2017). Especially that the dose–response relationship of HEV is unknown, and it is not clear which level of infectivity reduction is required to prevent infection in human. Therefore, the generation of more detailed data on the infectivity reduction for different HEV strain-matrix combinations would enhance our understanding of HEV stability in the environment and in foods (Johne et al., 2016).

Altogether, in the present study we have demonstrated that the effect of HPP on inactivation of HEV depends on the surrounding matrix. We have also observed an incomplete inactivation of HEV by HPP, which indicates that HPP treatment alone might not be sufficient in removing the risk of HEV contamination in high-risk commodities.

## Data Availability Statement

The datasets generated for this study can be found in the GenBank under Accession Numbers MN535994–MN535997.

## Author Contributions

NN performed the experimental design, data analysis, and wrote the manuscript. TD, AC, and JH conducted the experiments and data analysis. AG supervised the HPP experiments and assisted with manuscript preparation.

## Conflict of Interest

The authors declare that the research was conducted in the absence of any commercial or financial relationships that could be construed as a potential conflict of interest.

## References

[B1] Anonymous (2017). *Microbiology of the Food Chain – Horizontal Method for Determination of Hepatitis A Virus and Norovirus Using Real-Time RT-PCR – Part 1: Method for Quantification. ISO 15216-1:2017*. Geneva: International Organization for Standardization.

[B2] Balamurugan S. Inmanee P. Souza J. Strange P. Pirak T. Barbut S. (2018). Effects of high pressure processing and hot water pasteurization of cooked sausages on inactivation of inoculated listeria monocytogenes, natural populations of lactic acid bacteria, *Pseudomonas* spp., and coliforms and their recovery during storage at 4 and 10 degrees C. *J. Food Prot.* 81 1245–1251. 10.4315/0362-028X.JFP-18-024 29969296

[B3] Barnaud E. Rogee S. Garry P. Rose N. Pavio N. (2012). Thermal inactivation of infectious hepatitis E virus in experimentally contaminated food. *Appl. Environ. Microbiol.* 78 5153–5159. 10.1128/AEM.00436-12 22610436 PMC3416424

[B4] Bidawid S. Farber J. M. Sattar S. A. Hayward S. (2000). Heat inactivation of hepatitis A virus in dairy foods. *J. Food Prot.* 63 522–528. 10.4315/0362-028x-63.4.522 10772219

[B5] Bose P. D. Das B. C. Hazam R. K. Kumar A. Medhi S. Kar P. (2014). Evidence of extrahepatic replication of hepatitis E virus in human placenta. *J. Gen. Virol.* 95 1266–1271. 10.1099/vir.0.063602-0 24622580

[B6] Chauhan A. Webb G. Ferguson J. (2019). Clinical presentations of Hepatitis E: a clinical review with representative case histories. *Clin. Res. Hepatol. Gastroenterol.* 43 649–657. 10.1016/j.clinre.2019.01.005 30808575 PMC6864596

[B7] Cook N. D’Agostino M. Johne R. (2017). Potential approaches to assess the infectivity of hepatitis E virus in pork products: a review. *Food Environ. Virol.* 9 243–255. 10.1007/s12560-017-9303-7 28470455

[B8] Cook N. Knight A. Richards G. P. (2016). Persistence and elimination of human norovirus in food and on food contact surfaces: a critical review. *J. Food Prot.* 79 1273–1294. 10.4315/0362-028X.JFP-15-570 27357051

[B9] Cook N. van der Poel W. H. (2015). Survival and elimination of hepatitis E virus: a review. *Food Environ. Virol.* 7 189–194. 10.1007/s12560-015-9196-2 25989918

[B10] Edgar R. C. (2004). MUSCLE: multiple sequence alignment with high accuracy and high throughput. *Nucleic Acids Res.* 32 1792–1797. 10.1093/nar/gkh340 15034147 PMC390337

[B11] Emmoth E. Rovira J. Rajkovic A. Corcuera E. Wilches Perez D. Dergel I. (2017). Inactivation of viruses and bacteriophages as models for swine hepatitis E virus in food matrices. *Food Environ. Virol.* 9 20–34. 10.1007/s12560-016-9268-y 27783334

[B12] Fujiwara S. Yokokawa Y. Morino K. Hayasaka K. Kawabata M. Shimizu T. (2014). Chronic hepatitis E: a review of the literature. *J. Viral. Hepat.* 21 78–89. 10.1111/jvh.12156 24383921

[B13] Glitscher M. Himmelsbach K. Woytinek K. Johne R. Reuter A. Spiric J. (2018). Inhibition of hepatitis E virus spread by the natural compound silvestrol. *Viruses* 10:301. 10.3390/v10060301 29865243 PMC6024817

[B14] Guu T. S. Liu Z. Ye Q. Mata D. A. Li K. Yin C. (2009). Structure of the hepatitis E virus-like particle suggests mechanisms for virus assembly and receptor binding. *Proc. Natl. Acad. Sci. U.S.A.* 106 12992–12997. 10.1073/pnas.0904848106 19622744 PMC2722310

[B15] Hartl J. Wehmeyer M. H. Pischke S. (2016). Acute hepatitis E: two sides of the same coin. *Viruses* 8:299. 10.3390/v8110299 27827877 PMC5127013

[B16] Hirneisen K. A. Hoover D. G. Hicks D. T. Pivarnik L. F. Kniel K. E. (2012). Pressure inactivation of entteric viruses in a seafood salad-like product. *J. Aquat. Food Prod. Technol.* 21 455–467. 10.1080/10498850.2011.609636

[B17] Hugas M. Garriga M. Monfort J. M. (2002). New mild technologies in meat processing: high pressure as a model technology. *Meat Sci.* 62 359–371. 10.1016/s0309-1740(02)00122-5 22061612

[B18] Johne R. Reetz J. Ulrich R. G. Machnowska P. Sachsenroder J. Nickel P. (2014). An ORF1-rearranged hepatitis E virus derived from a chronically infected patient efficiently replicates in cell culture. *J. Viral. Hepat.* 21 447–456. 10.1111/jvh.12157 24750215

[B19] Johne R. Trojnar E. Filter M. Hofmann J. (2016). Thermal stability of hepatitis E virus as estimated by a cell culture method. *Appl. Environ. Microbiol.* 82 4225–4231. 10.1128/AEM.00951-16 27208095 PMC4959202

[B20] Kamar N. Abravanel F. Lhomme S. Rostaing L. Izopet J. (2015). Hepatitis E virus: chronic infection, extra-hepatic manifestations, and treatment. *Clin. Res. Hepatol. Gastroenterol.* 39 20–27. 10.1016/j.clinre.2014.07.005 25150374

[B21] King N. J. Hewitt J. Perchec-Merien A. M. (2018). Hiding in plain sight? It’s time to investigate other possible transmission routes for hepatitis E virus (HEV) in developed countries. *Food Environ. Virol.* 10 225–252. 10.1007/s12560-018-9342-8 29623595

[B22] Kingsley D. H. (2013). High pressure processing and its application to the challenge of virus-contaminated foods. *Food Environ. Virol.* 5 1–12. 10.1007/s12560-012-9094-9 23412716 PMC3590410

[B23] Kingsley D. H. Hoover D. G. Papafragkou E. Richards G. P. (2002). Inactivation of hepatitis A virus and a calicivirus by high hydrostatic pressure. *J. Food Prot.* 65 1605–1609. 10.4315/0362-028x-65.10.1605 12380746

[B24] Komora N. Bruschi C. Ferreira V. Maciel C. Brandao T. R. S. Fernandes R. (2018). The protective effect of food matrices on Listeria lytic bacteriophage P100 application towards high pressure processing. *Food Microbiol.* 76 416–425. 10.1016/j.fm.2018.07.002 30166169

[B25] Krog J. S. Larsen L. E. Breum S. O. (2019). Tracing hepatitis E virus in pigs from birth to slaughter. *Front. Vet. Sci.* 6:50. 10.3389/fvets.2019.00050 30873419 PMC6400844

[B26] Larkin M. A. Blackshields G. Brown N. P. Chenna R. McGettigan P. A. McWilliam H. (2007). Clustal W and clustal X version 2.0. *Bioinformatics* 23 2947–2948. 10.1093/bioinformatics/btm404 17846036

[B27] Leon J. S. Kingsley D. H. Montes J. S. Richards G. P. Lyon G. M. Abdulhafid G. M. (2011). Randomized, double-blinded clinical trial for human norovirus inactivation in oysters by high hydrostatic pressure processing. *Appl. Environ. Microbiol.* 77 5476–5482. 10.1128/AEM.02801-10 21705552 PMC3147477

[B28] Lou F. DiCaprio E. Li X. Dai X. Ma Y. Hughes J. (2016). Variable high-pressure-processing sensitivities for genogroup II human noroviruses. *Appl. Environ. Microbiol.* 82 6037–6045. 10.1128/AEM.01575-16 27474724 PMC5038020

[B29] Lou F. Ye M. Ma Y. Li X. DiCaprio E. Chen H. (2015). A gnotobiotic pig model for determining human norovirus inactivation by high-pressure processing. *Appl. Environ. Microbiol.* 81 6679–6687. 10.1128/AEM.01566-15 26187968 PMC4561694

[B30] Motoya T. Umezawa M. Goto K. Doi I. Nagata N. Ikeda Y. (2019). High prevalence of hepatitis E virus infection among domestic pigs in Ibaraki prefecture. *Japan. BMC Vet. Res.* 15:87. 10.1186/s12917-019-1816-x 30866949 PMC6416891

[B31] Murrison L. B. Sherman K. E. (2017). The enigma of hepatitis E virus. *Gastroenterol. Hepatol. (N Y)* 13 484–491. 28867980 PMC5572962

[B32] Mykytczuk O. Harlow J. Bidawid S. Corneau N. Nasheri N. (2017). Prevalence and molecular characterization of the hepatitis E virus in retail pork products marketed in Canada. *Food Environ. Virol.* 9 208–218. 10.1007/s12560-017-9281-9 28197972 PMC5429394

[B33] Nasheri N. Petronella N. Ronholm J. Bidawid S. Corneau N. (2017). Characterization of the genomic diversity of norovirus in linked patients using a metagenomic deep sequencing approach. *Front. Microbiol.* 8:73. 10.3389/fmicb.2017.00073 28197136 PMC5282449

[B34] Panda S. K. Varma S. P. (2013). Hepatitis e: molecular virology and pathogenesis. *J. Clin. Exp. Hepatol.* 3 114–124. 10.1016/j.jceh.2013.05.001 25755485 PMC3940135

[B35] Park W. J. Park B. J. Ahn H. S. Lee J. B. Park S. Y. Song C. S. (2016). Hepatitis E virus as an emerging zoonotic pathogen. *J. Vet. Sci.* 17 1–11. 10.4142/jvs.2016.17.1.1 27051334 PMC4808633

[B36] Pisano M. B. Martinez-Wassaf M. G. Mirazo S. Fantilli A. Arbiza J. Debes J. D. (2018). Hepatitis E virus in South America: the current scenario. *Liver Int.* 38 1536–1546. 10.1111/liv.13881 29788538

[B37] Primadharsini P. P. Nagashima S. Okamoto H. (2019). Genetic variability and evolution of hepatitis E virus. *Viruses Res.* 127 216–228. 10.1016/j.virusres.2007.02.002 31109076 PMC6563261

[B38] Qi Y. Zhang F. Zhang L. Harrison T. J. Huang W. Zhao C. (2015). Hepatitis E virus produced from cell culture has a lipid envelope. *PLoS One* 10:e0132503. 10.1371/journal.pone.0132503 26161670 PMC4498737

[B39] Randazzo W. Vasquez-Garcia A. Aznar R. Sanchez G. (2018). Viability RT-qPCR to Distinguish between HEV and HAV with intact and altered capsids. *Front. Microbiol.* 9:1973. 10.3389/fmicb.2018.01973 30210465 PMC6119771

[B40] Salines M. Andraud M. Rose N. (2017). From the epidemiology of hepatitis E virus (HEV) within the swine reservoir to public health risk mitigation strategies: a comprehensive review. *Vet. Res.* 48:31. 10.1186/s13567-017-0436-3 28545558 PMC5445439

[B41] Salines M. Demange A. Stephant G. Renson P. Bourry O. Andraud M. (2019). Persistent viremia and presence of hepatitis E virus RNA in pig muscle meat after experimental co-infection with porcine reproductive and respiratory syndrome virus. *Int. J. Food Microbiol.* 292 144–149. 10.1016/j.ijfoodmicro.2018.12.023 30599454

[B42] Schemmerer M. Apelt S. Trojnar E. Ulrich R. G. Wenzel J. J. Johne R. (2016). Enhanced replication of hepatitis E virus strain 47832c in an A549-derived subclonal cell line. *Viruses* 8:267. 10.3390/v8100267 27690085 PMC5086603

[B43] Smith D. B. Simmonds P. International Committee on Taxonomy of Viruses Hepeviridae Study Group Jameel S. Emerson S. U. Harrison T. J. (2014). Consensus proposals for classification of the family Hepeviridae. *J. Gen. Virol.* 95 2223–2232. 10.1099/vir.0.068429-0 24989172 PMC4165930

[B44] Sooryanarain H. Meng X. J. (2019). Hepatitis E virus: reasons for emergence in humans. *Curr. Opin. Virol.* 34 10–17. 10.1016/j.coviro.2018.11.006 30497051 PMC6476702

[B45] Tamura K. Stecher G. Peterson D. Filipski A. Kumar S. (2013). MEGA6: molecular evolutionary genetics analysis version 6.0. *Mol. Biol. Evol.* 30 2725–2729. 10.1093/molbev/mst197 24132122 PMC3840312

[B46] Van der Poel W. H. M. Dalton H. R. Johne R. Pavio N. Bouwknegt M. Wu T. (2018). Knowledge gaps and research priorities in the prevention and control of hepatitis E virus infection. *Transbound. Emerg. Dis.* 65(Suppl. 1), 22–29. 10.1111/tbed.12760 29318757

[B47] Williams T. P. Kasorndorkbua C. Halbur P. G. Haqshenas G. Guenette D. K. Toth T. E. (2001). Evidence of extrahepatic sites of replication of the hepatitis E virus in a swine model. *J. Clin. Microbiol.* 39 3040–3046. 10.1128/jcm.39.9.3040-3046.2001 11526125 PMC88293

