# Supplementary material for: Evaluation of High-Pressure Processing in Inactivation of the Hepatitis E Virus
Source: Front Microbiol. 2020 Mar 24;11:461. doi: 10.3389/fmicb.2020.00461 (PMC7105680; doi:10.3389/fmicb.2020.00461)
Supplement: Supplementary file 1 [file Data_Sheet_1.docx]

**Supplementary Table 1-** The ratio between the inoculated genomes and the harvested extracellular genomes at 14 days post-infection.

| **Inoculated genomes** | **Extracellular genomes** | **Ratio** |
| --- | --- | --- |
| 1.00E+06 | 7.32E+04 | 13.659 |
| 5.00E+05 | 5.47E+04 | 9.1489 |
| 1.00E+05 | 2.36E+04 | 4.2392 |
| 5.00E+04 | 8.38E+03 | 5.9678 |
| 2.50E+04 | 3.67E+03 | 6.8093 |
| 1.67E+04 | 1.75E+03 | 9.5114 |
| 1.25E+04 | 1.39E+03 | 8.9836 |
| 1.13E+04 | 7.31E+02 | 15.381 |
| 1.00E+04 | 5.58E+02 | 17.912 |
| Average |  | 10.179 |
| STDEV |  | 4.8 |

**Supplementary Table 2.** HEV genome quantification from infectivity assay post-treatment in growth medium

| **Matrix** |  | Media |  |  |  |  |  |
| --- | --- | --- | --- | --- | --- | --- | --- |
| **Sample** |  | A | B | C | D | Average | STDEV |
| **Untreated** | undiluted | 23598 | 21210.6 | 21804 | 33300 | 24978.15 | 5639.9664 |
|  | 1:10 diluted | 1876 | 5354.4 | 1352.4 | 5064.6 | 3411.85 | 2090.076 |
|  |  |  |  |  |  |  |  |
| **400 MPa (1 min)** | undiluted | 303.6 | 248.4 | 212.52 |  | 254.84 | 45.880244 |
|  | 1:10 diluted | 25.2 | 14.52 | 18.6 |  | 19.44 | 5.3893228 |
|  |  |  |  |  |  |  |  |
| **400 MPa (5 min)** | undiluted | 234.6 | 303.6 | 248.4 |  | 262.2 | 36.511368 |
|  | 1:10 diluted | 17.8 | 20.7 | 13.24 |  | 17.246667 | 3.760656 |
|  |  |  |  |  |  |  |  |
| **600 MPa (1 min)** | undiluted | 182.16 | 176.64 | 167.2 |  | 179.4 | 3.9032294 |
|  | 1:10 diluted | 18.92 | 18.4 |  |  | 18.66 | 0.3676955 |
|  |  |  |  |  |  |  |  |
| **600 MPa (5 min)** | undiluted | 99.36 | 102.12 | 149.04 |  | 116.84 | 27.920143 |
|  | 1:10 diluted | 20.7 | 15.8 | 27.6 |  | 21.366667 | 5.9281813 |

**Supplementary Table 3.** HEV genome quantification from infectivity assay post-treatment in pork pâté samples

| **Matrix** |  | pâté |  |  |  |  |  |
| --- | --- | --- | --- | --- | --- | --- | --- |
| **Sample** |  | A | B | C | D | Average | STDEV |
| **Untreated** | undiluted | 3576.96 | 3974.4 | 5593.6 |  | 4381.6533 | 1068.2231 |
|  | 1:10 diluted | 690 | 154.6 | 143.2 | 132.8 | 329.26667 | 273.37834 |
|  |  |  |  |  |  |  |  |
| **400 MPa (1 min)** | undiluted | 2944 | 2149.12 | 2472.96 |  | 2522.0267 | 399.70515 |
|  | 1:10 diluted |  |  |  |  |  |  |
|  |  |  |  |  |  |  |  |
| **400 MPa (5 min)** | undiluted | 1943.04 | 1501.44 | 1898.88 |  | 1781.12 | 243.21432 |
|  | 1:10 diluted |  |  |  |  |  |  |
|  |  |  |  |  |  |  |  |
| **600 MPa (1 min)** | undiluted | 2649.6 | 2031.36 | 2185.92 |  | 2288.96 | 321.7423 |
|  | 1:10 diluted |  |  |  |  |  |  |
|  |  |  |  |  |  |  |  |
| **600 MPa (5 min)** | undiluted | 2296.32 | 1192.32 | 1324.8 |  | 1604.48 | 602.80152 |
|  | 1:10 diluted |  |  |  |  |  |  |

**Supplementary Figure 1.** Visual assessment of HPP processed pork pâté samples. Samples were treated in triplicate at 400 MPa (A & B) and 600 MPa (C & D) for 1 min and 5 min respectively at ambient temperature. Visual inspection and documentation were conducted before and after treatments to detect any changes in food quality or appearance (size, colour, texture, and secretion of fluids).

**
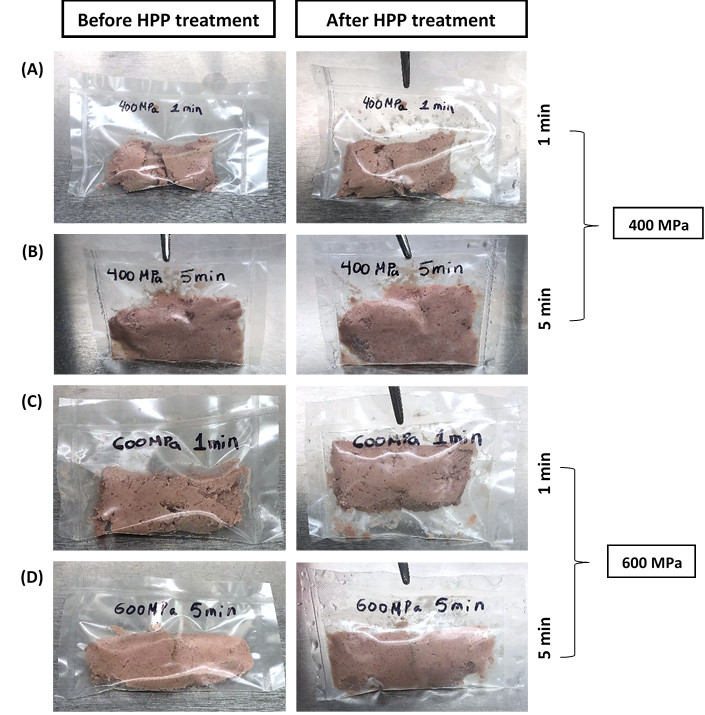
**
